# Silencing of PTX3 alleviates LPS-induced inflammatory pain by regulating TLR4/NF-κB signaling pathway in mice

**DOI:** 10.1042/BSR20194208

**Published:** 2020-02-04

**Authors:** Shuang Qi, Feng Zhao, Zinan Li, Feng Liang, Shanshan Yu

**Affiliations:** 1 Department of Anesthesiology, China-Japan Union Hospital of Jilin University, Changchun City, Jilin Province 130033, P.R. China; 2 Department of Operating Room, China-Japan Union Hospital of Jilin University, Changchun City, Jilin Province 130033, P.R. China

**Keywords:** inflammation, inflammatory pain, PTX3, TLR4/NF-κB axis

## Abstract

Pentraxin 3 (PTX3), an inflammatory marker and a pattern recognition receptor, plays an important role in promoting the progress of tumor and inflammatory diseases. However, the role of PTX3 in the pathogenesis of inflammatory pain diseases is rarely reported. The purpose of the present study is to investigate the effect of PTX3 on the progression of inflammatory pain and the special molecular mechanism. A mouse BV2 microglia cell activation-mediated inflammatory model was developed with Lipopolysaccharide (LPS) induction, and a mouse inflammatory pain model was established with LPS injection. The effect of PTX3 on microglia inflammatory activation was verified by measuring pro-inflammatory cytokines expression. The mechanical hyperalgesia testing, the thermal preference testing and the cold allodynia testing were used to measure the response of mice to mechanical pain, heat stimulation and cold stimulation, respectively. The results revealed that the expression of PTX3 was decreased in the LPS-induced inflammatory pain mice model. Silencing of PTX3 down-regulated LPS-induced inflammatory factors, including IL-6, NO and TNF-α, and alleviated LPS-induced inflammatory pain in BV2 cells. In addition, overexpression of TLR4 reversed the inhibitory effect of si-PTX3 on LPS-induced inflammatory response in BV2 cells. What is more, silencing of PTX3 inhibited TLR4/NF-κB signaling pathway. Collectively, it suggests that silencing of PTX3 alleviates LPS-induced inflammatory response of BV2 cells potentially by regulating the TLR4/NF-κB signaling pathway.

## Introduction

Inflammatory pain is a chronic inflammation caused by tissue damage and harmful stimulation. It is characterized by hyperalgesia and hyperalgesia in the injured area and adjacent tissues [1,2]. There are about 116 million people suffer from inflammatory pain per year in America [3]. Inflammatory pain affects the daily life of patients by changing their general functions and activities, often leading to motor dysfunction, and may also affect the prognosis of patients. At present, traditional analgesics, such as NSAIDs, are used to treat inflammatory pain [4]. However, this treatment is only partially effective, which may be accompanied by serious side effects and become a major clinical problem [5,6]. Therefore, it is of great significance to find new targets and effective strategies for the treatment of inflammatory pain.

Pentraxin 3 (PTX3) is an evolutionarily conserved pattern recognition molecule, which is produced by different cell responses to pro-inflammatory stimuli [7]. It activates effectors under inflammatory conditions and is an important component of innate immunity [8]. PTX3 expression is regulated by various signaling pathways, such as NF-κB, JNK and PI3K/Akt signaling pathways [9]. PTX3 was originally identified as the inducible genes of IL-1 and TNF-α, which are widely involved in the regulation of inflammatory diseases, such as pneumonia, cystitis and cancer-related inflammation [10]. In addition, PTX3 is involved in the occurrence and development of cancer and can be used as a marker of cancer progression [11,12]. It was found that the expression of PTX3 in low-grade and high-grade tumors was different and positively correlated with tumor grade and severity. Therefore, PTX3 may be a new marker of tumor related inflammation and malignant glioma [13]. However, at present, the conclusion about the function of PTX3 has not been formed in inflammatory pain. Therefore, it is still necessary to further explore its function and mechanism.

Toll-like receptor (TLR) is a kind of natural immune receptor. The moderate expression of TLR4 maintains the defense function of the body, and plays an important role in resisting and clearing the infection of pathogenic microorganism, and maintaining the steady state of the immune system of the body [14,15]. Activation of TLR4 signaling pathway induces the production of proinflammatory factors, leading to autoimmune diseases and inflammatory diseases [16]. Nuclear factor-kappa B (NF-κB) is involved in the response of cells to external stimulation, and contributes to in the process of inflammatory response and immune response [17]. The inappropriate regulation of NF-κB leads to autoimmune diseases, chronic inflammation and various cancers [18,19]. It is reported that PCSK9 silencing inhibits atherosclerotic vascular inflammation by inhibiting TLR4/NF-κB signaling pathway [20]. In addition, miR-223 alleviates LPS-induced lung injury inflammation by inhibiting TLR4/NF-κB signaling pathway [21].

In the present study, we explored the involvement of PTX3 in the inflammatory response and its underlying mechanisms using the LPS-induced inflammatory pain model *in vitro* and LPS-injected mouse model. Our results demonstrated that silencing of PTX3 played a significant role in cancellation inflammatory pain through regulating inflammatory response via the TLR4/NF-κB signaling pathway.

## Materials and methods

### Cell culture

The murine BV2 microglial cells were purchased from the American Type Culture Collection (ATCC, Manassas, VA). The cell was cultured in DMEM, 10% fetal bovine serum (Gibco), 1% glutamine, 100 U/ml penicillin sodium, 100 μg/ml streptomycin sulfate (Sigma) at 37°C, 5% CO_2_. When the cell number reached 60–70%, it was washed with PBS for standby.

### Animals

Male Swiss mice (20–25 g) (*n* = 10 per group/4 groups) were used. All mice were housed in a temperature and humidity-controlled room (22–25°C, 55–60%). All mice were acclimatized for 1 week and free access to food and water in a 12 h light/dark cycle. To establish the inflammatory pain model, mice were injected with LPS (200 ng in 25 μl of sterile saline) in their right hind paw. The mice that were injected with the same volume of saline was a control group. The animals were killed with pentobarbitone (100 mg/kg). All experiments were performed in China-Japan Union Hospital of Jilin University and approved by the Ethics Committee of China-Japan Union Hospital of Jilin University.

### Reagents

The following reagents were used: The BV2 cells (San Diego, CA, U.S.A.); GAPDH, anti-PTX3-1, anti-TLR4, anti-COX-2, anti-iNOS and anti-p65 (Wuhan Sanying Biotechnology Co., Ltd., Wuhan, China); DMEM (Gibco, Carlsbad, CA, U.S.A.); RNA extraction kit, reverse transcription kit, RT-PCR Kit (Invitrogen, Carlsbad, CA, U.S.A.), primer synthesis (Takara, Dalian, China), protein quantitative kit, cell lysate (Biyuntian Biotechnology Research Institute, Nantong City, China).

### Behavioral tests

At least 3 days before operation and 1, 3, 5 and 7 days after injection were predicted. Von Frey FL was used in the mechanical hyperalgesia test. A series of increasing pressures were applied to the back paws of mice. Apply pressure for 5–6 s, 10 times each. The mechanical withdrawal threshold (MWT) was used as a record of claw withdrawal. For thermal preference testing, radiant heat was placed under the sole of the hind paw. In order to avoid tissue damage, the hind legs were removed from the heat source every 40 s and the thermal retreat latency (TRL) was recorded. In the cold pain test, use a syringe attached to the polyethylene tube to gently drop a drop of acetone onto each rear paw. Rapid claw contraction is thought to be a manifestation of cold hyperalgesia. Repeat the test for three times, and the interval between each test is 5–10 min.

### Transfection

si-PTX3, Ctrl-siRNA, pcDNA3.1 and pcDNA3.1-TLR4 were designed and synthesized by Tsingke Biotech Co., Ltd. (Beijing, China). BV2 cells were inoculated into six-well culture plate with a density of 1×10^5^ cells/well. Transfection assay according to the instructions of cell transfection Kit.

### ELISA

The TNF-α (BioSite, Paris, France) and IL-6 (BioSite, Paris, France) concentrations in the supernatants were detected using corresponding ELISA kits according to the instructions.

### Nitrite oxide production assay

The supernatants of LPS-treated microglial BV2 cells were collected and the content of nitrite oxide (NO) was examined by Nitric Oxide assay kit (Thermo Fisher Scientific, Massachusetts, U.S.A.).

### RT-qPCR

Total RNA samples from the BV2 cells were isolated using TRIzol^®^ reagent (Invitrogen, Carlsbad, CA, U.S.A.). Using specific miRNA RT primers to the reverse transcription reaction (Invitrogen, Carlsbad, CA, U.S.A.). The thermocycling conditions of RT-qPCR were as follows: 95°C for 1min; 30 cycles of 94°C for 30 s, 55°C for 20 s and 72°C for 15 s. Relative transcriptional levels were calculated by the 2^–ΔΔCT^ method with GAPDH as a normalizing gene.

### Western blotting

The cells were inoculated into six-well plates, 1×10^4^ cells/well, and the supernatant was removed 24 h later. The plasmid was transfected and the cells were collected 48 h. The protein extracted from the cells was collected. Cells were lysed with improved Ripa buffer (Sigmag–Aldrich), and the protein content was measured by Bradford reagent (Thermo Scientific). The extracted protein (50 μg) was separated from denatured polyacrylamide Gel and then transferred to PVDF membrane (microporous), sealed with 5% skim milk (HiMedia). Then, the enhanced laboratories (ECL) darkroom development, Bio-Rad Laboratories (California, U.S.A.) scan record, and anti-GAPDF as internal reference were used for analysis and comparison.

### Statistical analyses

Data are represented as means±SD and each experiment was performed in triplicate in the present study. One-way ANOVA and Student’s unpaired *t* test were used to analyze statistical significance. All statistical analyses were performed by SPSS 20.0 software (SPSS, Inc., Chicago, IL, U.S.A.). *P*-value < 0.05 were considered to be significant.

## Results

### LPS-induced inflammatory pain was relieved in PTX3 knockout mice

**Figure 1:**
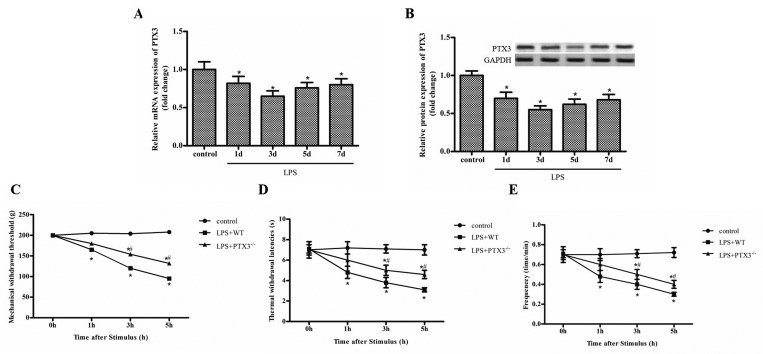
LPS-induced inflammatory pain is relieved in PTX3 knockout mice (**A**) The mRNA expression of PTX3 in mice treated with LPS (200 ng in 25 μl of sterile saline) at 1, 3, 5 and 7 days. (**B**) The protein expression of PTX3 in mice treated with LPS (200 ng in 25 μl of sterile saline) at 1, 3, 5 and 7 days. (**C**) MWT after injection. (**D**) Thermal withdrawal latencies after injection; (**E**) Frequency responses to cold stimulation after injection. ‘*’ means compared with the control group at *P<*0.05, and ‘#’ means compared with the LPS+Normal group at *P*<0.05. GAPDH was used as an invariant internal control for calculating protein fold changes.

We established the inflammatory pain model through injecting LPS into mice hind paw, including PTX3 knockout mice and WT mice. Firstly, we analyzed the expression of PTX3 by qRT-PCR in the WT mice model. As shown in Figure 1A, the expression of PTX3 on 1, 3, 5 and 7 days after injection of LPS was significantly decreased compared with the control group, and the lowest on 3 day after injection of LPS (Figure 1A,B). More importantly, we explored the effect of PTX3 on paw retraction after mechanical, hot and cold stimulation. We found that LPS significantly reduces the mice to the frequency response of MWTs, thermal withdrawal latencies (TWLs) and cold stimulation compared with control group, while MWTs, TWLs and the frequency response of cold stimulation were significantly increased in PTX3 knockout mice compared with normal mice (Figure 1C–E). These results suggest that silencing of PTX3 alleviated LPS induced inflammatory pain in PTX3^−/−^ mice.

### Silencing of PTX3 alleviates LPS-induced inflammatory response in BV2 cells

**Figure 2:**
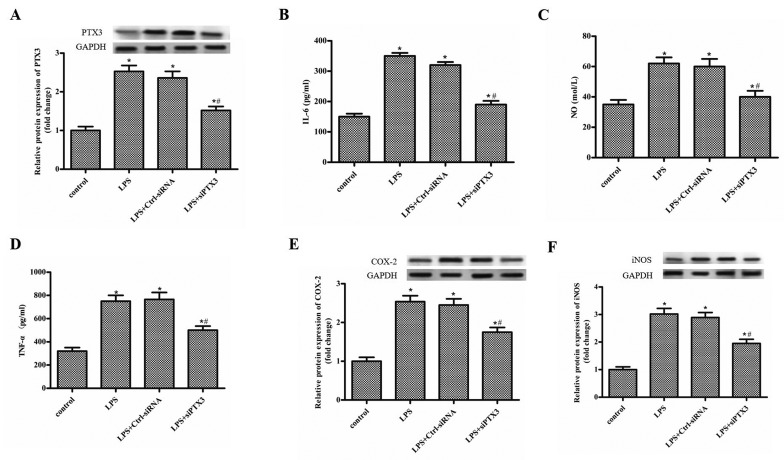
Silencing of PTX3 alleviates LPS-induced inflammatory response in BV2 cells The BV2 cells were treated with LPS (1 µg/ml), and transfected with Ctrl-siRNA, si-PTX3 respectively. and the levels of pro-inflammatory factors were detected by ELISA and Western blot. (**A**) The protein expression of PTX3 in the control, LPS, Ctrl-siRNA and si-PTX3 group. (**B**-**D**) The level of IL-6, NO and TNF-α in the control, LPS, Ctrl-siRNA and si-PTX3 group. (**E,F**) The protein expression of COX-2 and iNOS in the control, LPS, Ctrl-siRNA and si-PTX3 group. ‘*’ means compared with the control group at *P<*0.05, and ‘#’ means compared with the LPS+Ctrl-siRNA group at *P*<0.05. GAPDH was used as an invariant internal control for calculating protein fold changes.

The BV2 cells were treated with LPS (1 µg/ml), and transfected with Ctrl-siRNA, si-PTX3, respectively, and the levels of pro-inflammatory factors were detected by ELISA and Western blot. As shown in Figure 2A, PTX3 expression was significantly up-regulated after LPS treatment, while si-PTX3 inhibited the increase in PTX3 expression (Figure 2A). The levels of IL-6, NO and TNF-α were significantly increased after LPS treatment compared with the control group, while si-PTX3 significantly decreased the level of IL-6, NO and TNF-α compared with the LPS+Ctrl-siRNA group (Figure 2B–D). In addition, the expression of COX-2 and iNOS was increased after LPS treatment, while si-PTX3 decreased the expression of COX-2 and iNOS (Figure 2E,F).

### Silencing of PTX3 inhibits TLR4/NF-κB signaling pathway

**Figure 3:**
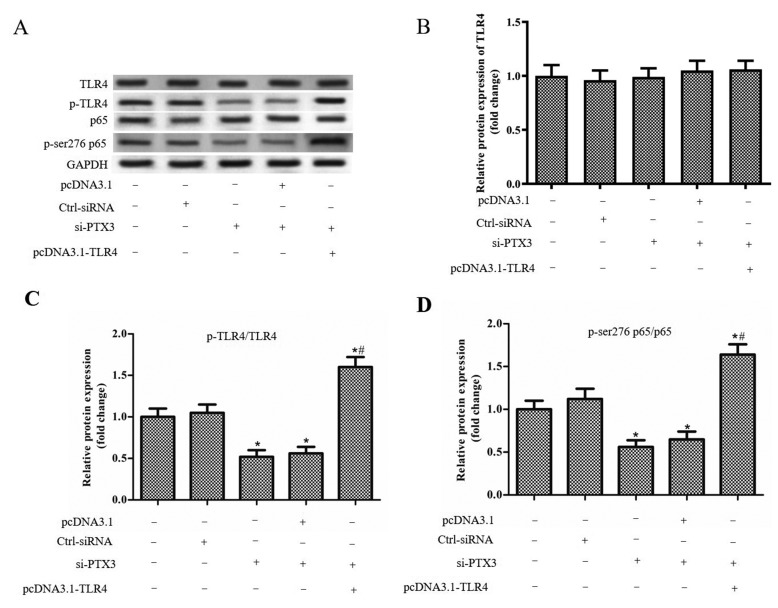
Silencing of PTX3 inhibits TLR4/NF-κB signaling pathway The BV2 cells were treated with LPS (1 µg/ml), and transfected with Ctrl-siRNA, si-PTX3, si-PTX3+pcDNA3.1 and si-PTX3+pcDNA3.1-TLR4, respectively. (**A**) Western blot was performed to confirm the protein expression levels of TLR4, p-TLR4, NF-κB, p65 and p-p65. (**B**) The protein expression of TLR4 in each group. (**C**) The protein expression of p-TLR4/TLR4 in each group. (**D**) The protein expression of p-p65/p65 in each group. ‘*’ means compared with the untreated group at *P*<0.05, and ‘#’ means compared with the si-PTX3+pcDNA3.1 group at *P*<0.05. GAPDH was used as an invariant internal control for calculating protein fold changes.

The BV2 cells were treated with LPS (1 µg/ml), and transfected with Ctrl-siRNA, si-PTX3, si-PTX3+pcDNA3.1 and si-PTX3+pcDNA3.1-TLR4, respectively. The results showed si-PTX3 and pcDNA3.1-TLR4 had no significant effect on the expression of TLR4 and NF-κB (Figure 3A–C). However, the levels of p-TLR4/TLR4 and p-p65/p65 were significantly decreased when transfected with si-PTX3, and co-transfection of si-PTX3 and pcDNA3.1-TLR4 promoted the expression levels of p-TLR4/TLR4 and p-p65/p65 compared with the si-PTX3+pcDNA3.1 group (Figure 3B–D). These results suggested that si-PTX3 inhibited the activity of TLR4/NF-κB signaling pathway.

### Overexpression of TLR4 reverses the inhibitory effect of si-PTX3 on inflammatory response in BV2 cells

**Figure 4:**
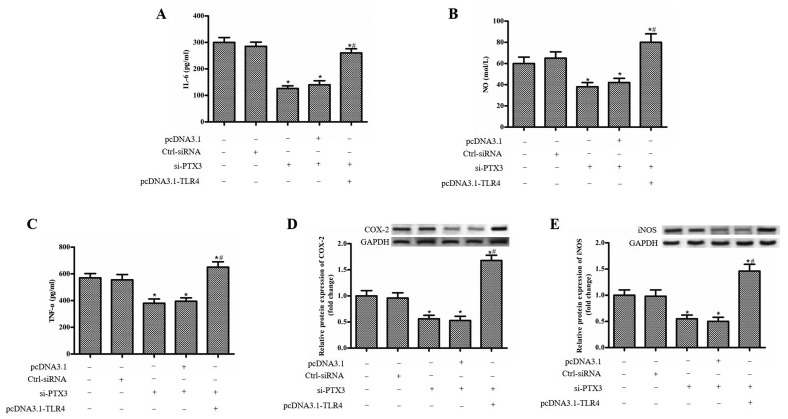
Overexpression of TLR4 reverses the inhibitory effect of si-PTX3 on inflammatory response in BV2 cells The BV2 cells were treated with LPS (1 µg/ml), and transfected with Ctrl-siRNA, si-PTX3, si-PTX3+pcDNA3.1 and si-PTX3+pcDNA3.1-TLR4. (**A**) The level of IL-6 in each group. (**B,A**) The level of NO in each group. (**C**) The level of TNF-α in each group. (**D**) The protein expression of COX-2 in each group. (**E**) The protein expression of iNOS in each group. ‘*’ means compared with the untreated group at *P*<0.05, and ‘#’ means compared with the si-PTX3+pcDNA3.1 group at *P*<0.05. GAPDH was used as an invariant internal control for calculating protein fold changes.

We next examined the role of TLR4 in inflammatory response of BV2 cells. Overexpression of TLR4 up-regulated the level of IL-6, NO and TNF-α compared with si-PTX3+pcDNA3.1 group (Figure 4A–C). What’s more, pcDNA3.1-TLR4 significantly up-regulated the protein expression of COX-2 and iNOS than the control and si-PTX3+pcDNA3.1 group (Figure 4D,E).

## Discussion

It is worth noting that microglia, macrophages in the central nervous system, plays an important role in neuroinflammation and inflammatory pain. Recent studies have shown that many adverse stimuli, including LPS, lead to the activation of microglia in the spinal cord. Activated microglia produce marker proteins and maintain cytotoxicity, which is important for inflammatory pain [22,23]. In the present research, we suggested that silencing of PTX3 alleviated LPS-induced inflammatory response by targeting TLR4 in BV2 cells. Furthermore, silencing of PTX3 inhibited the TLR4/NF-κB signaling pathway. It suggests that si-PTX3 alleviates LPS-induced inflammatory pain potentially by regulating the TLR4/NF-κB signaling pathway. Therefore, silencing of PTX3 may be a new treatment for inflammatory pain.

PTX3 is initially considered as a marker of inflammatory response, and it is involved in the occurrence and development of a variety of inflammatory diseases, including inflammatory related tumors, cardiovascular and cerebrovascular diseases, and neuroinflammation [24,25]. As we all know, cancer is closely related to inflammation, which has been widely accepted. It was found that PTX3 expression was significantly increased in various tumors, including lung cancer, pancreatic cancer, glioma and breast cancer [26–28]. Furthermore, a recent study found that, PTX3 caused endothelial dysfunction and damaged vascular system by inducing inflammatory response and metabolic changes of endothelial cells [29]. More interestingly, PTX3, as the target of CEBPD, weakens macrophage mediated phagocytosis in astrocytes. Therefore, PTX3 is considered to participate in the development of AD [30]. Likewise, in the present research, our research shown that silencing of PTX3 inhibits LPS induced inflammatory response, which is consistent with previous research conclusions. Studies have shown that when adverse stimuli, such as local inflammatory response, are introduced from the surrounding, the threshold of pain receptors is reduced, resulting in persistent pain [31]. Therefore, we further studied the effect of PTX3 knockout on inflammatory pain in LPS injected mice, which is a common inflammatory pain model [32]. Interestingly, PTX3 knockout significantly reduced MWTs and TWLs induced by LPS injection. In addition, it can also reduce the increase in pro-inflammatory cytokines induced by LPS.

Herein, we subsequently examined the mechanisms of PTX3 in inflammatory pain. We found that silencing of PTX3 can reduce the expression and secretion of TLR4 and NF-κB in LPS induced BV2 microglia. TLR4, a natural immune receptor, is often involved in inflammation related diseases. NF-κB family of proteins do regulate neuronal development and it is possible that there is involvement of NF-κB in regulating pain via direct mechanism like has been shown in case of regulation of food intake and energy expenditure. It has been reported that Rap1-mediated NF-κB activity regulates the paracrine capacity of mesenchymal stem cells in heart repair following infarction [33]. Coincidentally, Shi et al. [34] found that activation of NF-κB promotes pro-opiomelanocortin (POMC) protein expression, participation and chronicity. It has been reported that TLR4/NF-κB signaling pathway participates in the regulation of various inflammatory diseases. TLR4/NF-κB signaling pathway is involved in the regulation of PCSK9 on atherosclerosis inflammation [35]. Analogously, TLR4/NF-κB signaling pathway is one of the main inflammatory pathways, and its expression is inhibited by miR-146a, thus protecting human retinal microvascular endothelial cells [36]. Our results showed that overexpression of TLR4 increases levels of proinflammatory cytokines, including IL-6, NO, TNF-α, COX-2 and iNOS. As expected, si-PTX3-antagonized IL-6, TNF-α, and IL-1β generation were offset by TLR4 overexpression.

Inflammatory signaling underlies many diseases, from arthritis to cancer [37]. Shin et al. [38] found that a positive DP103/NF-κB feedback loop promotes constitutive NF-κB activation in invasive breast cancers and activation of this pathway is linked to cancer progression and the acquisition of chemotherapy resistance. Recently, Liu and co-workers [39] reported that the p52 transcription factor driven by noncanonical NF-κB signaling cooperates with ETS1/2 to regulate TERT expression specifically from the C250T-mutant promoter in glioblastoma. Activation and inflammatory responses of microglia are usually associated with TLR4/NF-κB signaling pathways, which in turn trigger a range of neurological diseases, such as hypothalamic inflammation [40], brain injury [41] and idiopathic Parkinson’s disease (IPD) [42]. Our findings showed that the activation of TLR4/NF-κB signaling was abrogated by si-PTX3 in LPS-activated BV2 cells. Moreover, TLR4 overexpression markedly counteracted the inhibition of si-PTX3 on TLR4/NF-κB phosphorylation. More interestingly, TLR4 overexpression also reversed si-PTX3-inhibited pro-inflammatory cytokines in LPS-activated BV2 cells. Therefore, inhibiting PTX3/TLR4/NF-κB pathway may be an effective treatment for inflammatory pain.

In summary, we found that silencing of PTX3 mitigated LPS-induced pain hypersensitivity and inflammation *in vivo*. Furthermore, silencing of PTX3 inhibited the LPS-induced TLR4, p-p65/p65 and NF-κB expression. Notably, silencing of PTX3 relieves LPS induced inflammatory response through the TLR4/NF-κB signaling pathway in BV2 cells. The results suggest that si-PTX3 has anti-inflammatory effect during the formation of inflammatory pain, and the molecular therapy of PTX3 provides a theoretical basis.

## Abbreviations

MWT: mechanical withdrawal threshold
NF-κB: nuclear factor-kappa B
PTX3: pentraxin 3
TLR: Toll-like receptor
TWL: thermal withdrawal latency
